# (*E*)-2,4,6-Trimethyl-*N*-[(1*H*-pyrrol-2-yl)methyl­idene]aniline

**DOI:** 10.1107/S160053681205057X

**Published:** 2012-12-19

**Authors:** Wolfgang Imhof

**Affiliations:** a University Koblenz-Landau, Institute for Integrated Natural Sciences, Universitätsstrasse 1, 56070 Koblenz, Germany

## Abstract

The title compound, C_14_H_16_N_2_, is a pyrrole-2-carbaldimine ligand that shows an *E* conformation at the imine double bond. The dihedral angle between the rings is 78.3 (1)°. In the crystal, pairs of mol­ecules form centrosymmetric dimers [graph-set descriptor is presumably *R* ^2^_2_(10)] *via* N—H⋯N hydrogen bonds between the pyrrole N—H group and the imine N atom of a neighbouring mol­ecule.

## Related literature

For structure analyses of other pyrrole-2-carbaldimines in which the substituents at the imine N atoms do not include functional groups that are capable of forming additional hydrogen bonds, see: Gomes *et al.* (2010 ▶); Crestani *et al.* (2011 ▶); Matsui *et al.* (2004 ▶); Wang *et al.* (2007 ▶); Franceschi *et al.* (2001 ▶); Tahir *et al.* (2010 ▶); Munro *et al.* (2006 ▶). For standard bond lengths, see: Allen *et al.* (1987 ▶). For graph-set description, see: Bernstein *et al.* (1995 ▶).
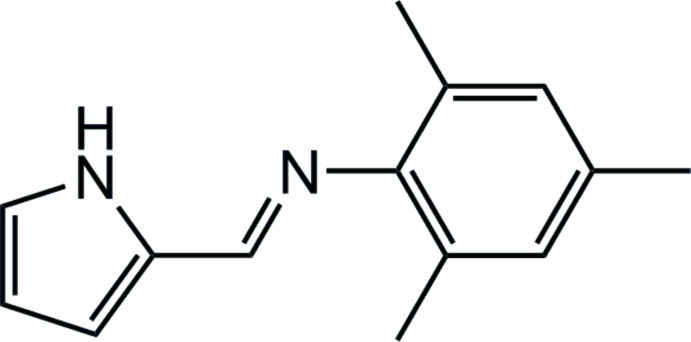


## Experimental

### 

#### Crystal data

C_14_H_16_N_2_
*M* *_r_* = 212.29
Monoclinic, 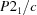

*a* = 13.6739 (10) Å
*b* = 7.3086 (6) Å
*c* = 13.3880 (11) Å
β = 111.184 (4)°
*V* = 1247.54 (17) Å^3^
*Z* = 4
Mo *K*α radiation
μ = 0.07 mm^−1^
*T* = 183 K
0.6 × 0.4 × 0.01 mm

#### Data collection

Nonius KappaCCD diffractometer
4714 measured reflections
2849 independent reflections
1346 reflections with *I* > 2σ(*I*)
*R*_int_ = 0.049

#### Refinement

*R*[*F* ^2^ > 2σ(*F* ^2^)] = 0.052
*wR*(*F* ^2^) = 0.127
*S* = 0.87
2849 reflections
152 parameters
H atoms treated by a mixture of independent and constrained refinement
Δρ_max_ = 0.16 e Å^−3^
Δρ_min_ = −0.26 e Å^−3^

### 

Data collection: *COLLECT* (Nonius, 1998 ▶); cell refinement: *DENZO* (Otwinowski & Minor, 1997 ▶); data reduction: *DENZO*; program(s) used to solve structure: *SHELXS97* (Sheldrick, 2008 ▶); program(s) used to refine structure: *SHELXL97* (Sheldrick, 2008 ▶); molecular graphics: *ORTEP-3* (Farrugia, 2012 ▶); software used to prepare material for publication: *publCIF* (Westrip, 2010 ▶).

## Supplementary Material

Crystal structure: contains datablock(s) I, global. DOI: 10.1107/S160053681205057X/bt6872sup1.cif

Structure factors: contains datablock(s) I. DOI: 10.1107/S160053681205057X/bt6872Isup2.hkl

Supplementary material file. DOI: 10.1107/S160053681205057X/bt6872Isup3.cml

Additional supplementary materials:  crystallographic information; 3D view; checkCIF report

## Figures and Tables

**Table 1:** Hydrogen-bond geometry (Å, °)

*D*—H⋯*A*	*D*—H	H⋯*A*	*D*⋯*A*	*D*—H⋯*A*
N1—H1*A*⋯N2^i^	0.94 (2)	2.05 (2)	2.909 (2)	150.7 (17)

Symmetry code: (i) 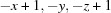.

**Fig. 1.**
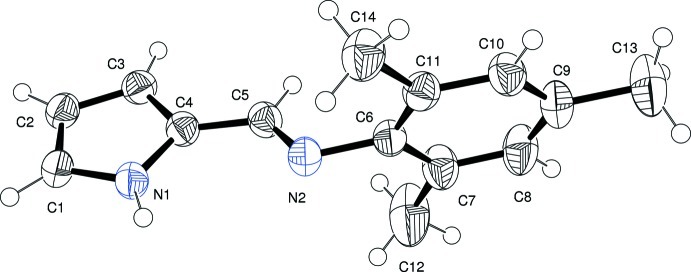
Molecular structure of the title compound with thermal ellipsoids at the 50% probability level.

**Fig. 2.**
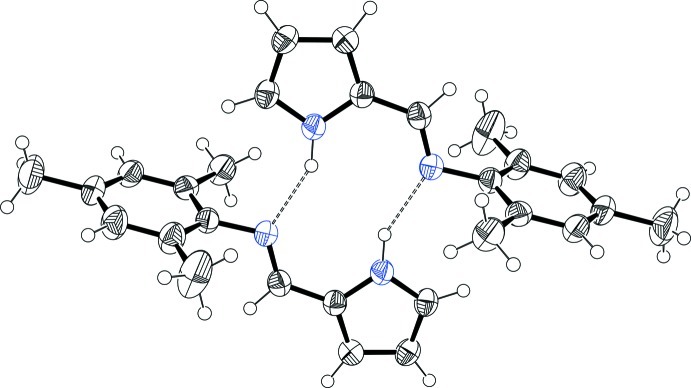
Centrosymmetric dimer of two molecules of the title compound connected by mutual N–H···N hydrogen bonds (symm code = 1 - x, -y, 1 - z).

## supplementary crystallographic information

### Comment

In the course of a project related to the supramolecular structures of square planar nickel and palladium complexes of pyrrole-2-carbaldehyde based Schiff base ligands in comparison with the structures of the free ligands the molecular structure of the title compound was determined. The free ligands form centrosymmetric dimers *via* N—H···N hydrogen bonds between the pyrrole NH function and the imine nitrogen atom of a neighboring molecule (Crestani *et al.*, 2011; Gomes *et al.*, 2010; Matsui *et al.*, 2004; Wang *et al.*, 2007; Franceschi *et al.*, 2001; Tahir *et al.*, 2010; Munro *et al.*, 2006).

The molecular structure of the title compound is depicted in Figure 1. The C—N imine double bond shows an *E*-configuration. All bond lengths correspond to expected values (Allen *et al.*, 1987). In Figure 2 the centrosymmetric dimer that is produced by two N—H···N hydrogen bonds between the pyrrole NH functions and the imine nitrogen atoms of a neighboring molecule is presented. Corresponding hydrogen bond parameters are summarized in Table 1.

### Experimental

Pyrrol-2-carbaldehyde (400 mg, 5 mmol) and 2,4,6-trimethylaniline (680 mg, 7 mmol) were dissolved in 20 ml anhydrous ethanol in the presence of 10 mg *p*-toluenesulfonic acid and the reaction mixture stirred at room temperature. The progress of the reaction was monitored by TLC. After the aldehyde was consumed completely the solution was cooled down to 4°C which led to the formation of crystalline material after 2 days (yield: 890 mg, 84%).

### Refinement

Carbon bound hydrogen atoms have been included into the refinement in calculated positions with fixed thermal parameter of *U*_iso_(H) = 1.2 *U*_eq_(C) for aromatic C—H groups and the imine C—H function and a thermal parameter of *U*_iso_(H) = 1.5 *U*_eq_(C) for methyl groups. The nitrogen bound hydrogen atom H1A has been detected from difference Fourier maps and was freely refined.

### Crystal data

**Table:**

C_14_H_16_N_2_	*F*(000) = 456
*M**_r_* = 212.29	*D*_x_ = 1.130 Mg m^−^^3^
Monoclinic, *P*2_1_/*c*	Mo *K*α radiation, λ = 0.71073 Å
Hall symbol: -P 2ybc	Cell parameters from 4714 reflections
*a* = 13.6739 (10) Å	θ = 3.1–27.5°
*b* = 7.3086 (6) Å	µ = 0.07 mm^−^^1^
*c* = 13.3880 (11) Å	*T* = 183 K
β = 111.184 (4)°	Plate, light yellow
*V* = 1247.54 (17) Å^3^	0.6 × 0.4 × 0.01 mm
*Z* = 4	

### Data collection

**Table:**

Nonius KappaCCD diffractometer	1346 reflections with *I* > 2σ(*I*)
Radiation source: fine-focus sealed tube	*R*_int_ = 0.049
Graphite monochromator	θ_max_ = 27.5°, θ_min_ = 3.1°
phi–scan, ω–scan	*h* = −17→17
4714 measured reflections	*k* = −8→9
2849 independent reflections	*l* = −17→17

### Refinement

**Table:**

Refinement on *F*^2^	Primary atom site location: structure-invariant direct methods
Least-squares matrix: full	Secondary atom site location: difference Fourier map
*R*[*F*^2^ > 2σ(*F*^2^)] = 0.052	Hydrogen site location: inferred from neighbouring sites
*wR*(*F*^2^) = 0.127	H atoms treated by a mixture of independent and constrained refinement
*S* = 0.87	*w* = 1/[σ^2^(*F*_o_^2^) + (0.0585*P*)^2^] where *P* = (*F*_o_^2^ + 2*F*_c_^2^)/3
2849 reflections	(Δ/σ)_max_ = 0.032
152 parameters	Δρ_max_ = 0.16 e Å^−^^3^
0 restraints	Δρ_min_ = −0.26 e Å^−^^3^

### Special details

**Table:**

Geometry. All e.s.d.'s (except the e.s.d. in the dihedral angle between two l.s. planes) are estimated using the full covariance matrix. The cell e.s.d.'s are taken into account individually in the estimation of e.s.d.'s in distances, angles and torsion angles; correlations between e.s.d.'s in cell parameters are only used when they are defined by crystal symmetry. An approximate (isotropic) treatment of cell e.s.d.'s is used for estimating e.s.d.'s involving l.s. planes.
Refinement. Refinement of *F*^2^ against ALL reflections. The weighted *R*-factor *wR* and goodness of fit *S* are based on *F*^2^, conventional *R*-factors *R* are based on *F*, with *F* set to zero for negative *F*^2^. The threshold expression of *F*^2^ > σ(*F*^2^) is used only for calculating *R*-factors(gt) *etc*. and is not relevant to the choice of reflections for refinement. *R*-factors based on *F*^2^ are statistically about twice as large as those based on *F*, and *R*- factors based on ALL data will be even larger.

### Fractional atomic coordinates and isotropic or equivalent isotropic displacement parameters (Å^2^)

**Table:**

	*x*	*y*	*z*	*U*_iso_*/*U*_eq_	
N1	0.40090 (11)	0.0272 (2)	0.34857 (13)	0.0391 (4)	
H1A	0.4178 (15)	−0.013 (3)	0.4198 (17)	0.054 (6)*	
C1	0.30475 (14)	0.0020 (2)	0.27045 (15)	0.0410 (5)	
H1	0.2480	−0.0638	0.2783	0.049*	
C2	0.30352 (14)	0.0875 (3)	0.17859 (15)	0.0419 (5)	
H2	0.2465	0.0917	0.1119	0.050*	
C3	0.40204 (13)	0.1670 (2)	0.20194 (15)	0.0394 (5)	
H3	0.4241	0.2353	0.1535	0.047*	
C4	0.46186 (13)	0.1297 (2)	0.30736 (14)	0.0362 (4)	
C5	0.56546 (13)	0.1952 (2)	0.36830 (15)	0.0383 (5)	
H5	0.6014	0.2614	0.3309	0.046*	
N2	0.61285 (11)	0.1715 (2)	0.46863 (12)	0.0392 (4)	
C6	0.71568 (13)	0.2505 (3)	0.51684 (14)	0.0379 (5)	
C7	0.80272 (14)	0.1717 (3)	0.50284 (16)	0.0499 (5)	
C8	0.90023 (15)	0.2513 (3)	0.55354 (17)	0.0573 (6)	
H8	0.9594	0.1983	0.5435	0.069*	
C9	0.91517 (14)	0.4035 (3)	0.61769 (16)	0.0505 (5)	
C10	0.82766 (14)	0.4780 (3)	0.63231 (15)	0.0460 (5)	
H10	0.8361	0.5824	0.6770	0.055*	
C11	0.72809 (13)	0.4035 (3)	0.58312 (14)	0.0395 (5)	
C12	0.79212 (18)	0.0021 (4)	0.4355 (2)	0.0873 (9)	
H12A	0.8620	−0.0459	0.4456	0.131*	
H12B	0.7555	0.0328	0.3598	0.131*	
H12C	0.7520	−0.0905	0.4572	0.131*	
C13	1.02310 (17)	0.4870 (4)	0.6716 (2)	0.0771 (8)	
H13A	1.0601	0.4888	0.6211	0.116*	
H13B	1.0630	0.4138	0.7344	0.116*	
H13C	1.0159	0.6123	0.6941	0.116*	
C14	0.63527 (15)	0.4899 (3)	0.60012 (18)	0.0559 (6)	
H14A	0.5910	0.5509	0.5340	0.084*	
H14B	0.6600	0.5800	0.6581	0.084*	
H14C	0.5945	0.3951	0.6193	0.084*	

### Atomic displacement parameters (Å^2^)

**Table:**

	*U*^11^	*U*^22^	*U*^33^	*U*^12^	*U*^13^	*U*^23^
N1	0.0332 (8)	0.0432 (9)	0.0382 (10)	−0.0029 (7)	0.0095 (8)	0.0026 (8)
C1	0.0302 (10)	0.0436 (11)	0.0455 (12)	0.0006 (8)	0.0092 (9)	0.0005 (9)
C2	0.0357 (10)	0.0418 (11)	0.0418 (12)	0.0019 (9)	0.0060 (9)	0.0000 (9)
C3	0.0393 (10)	0.0381 (10)	0.0418 (11)	0.0023 (9)	0.0159 (9)	0.0023 (9)
C4	0.0340 (10)	0.0352 (10)	0.0405 (11)	0.0001 (8)	0.0147 (9)	−0.0028 (8)
C5	0.0357 (10)	0.0370 (11)	0.0443 (12)	−0.0014 (8)	0.0170 (9)	−0.0024 (8)
N2	0.0332 (8)	0.0446 (9)	0.0385 (10)	−0.0026 (7)	0.0113 (7)	−0.0005 (7)
C6	0.0332 (10)	0.0403 (11)	0.0382 (11)	−0.0028 (8)	0.0103 (9)	0.0026 (9)
C7	0.0408 (11)	0.0517 (13)	0.0562 (13)	−0.0030 (10)	0.0164 (10)	−0.0150 (10)
C8	0.0330 (11)	0.0726 (15)	0.0652 (15)	0.0010 (10)	0.0165 (10)	−0.0113 (12)
C9	0.0353 (11)	0.0625 (14)	0.0476 (12)	−0.0104 (10)	0.0076 (10)	−0.0065 (11)
C10	0.0437 (12)	0.0471 (12)	0.0424 (12)	−0.0065 (9)	0.0098 (9)	−0.0059 (9)
C11	0.0384 (10)	0.0433 (11)	0.0362 (11)	0.0006 (9)	0.0126 (9)	0.0008 (9)
C12	0.0499 (14)	0.0880 (19)	0.117 (2)	0.0022 (13)	0.0213 (15)	−0.0536 (17)
C13	0.0420 (12)	0.0964 (19)	0.0839 (18)	−0.0194 (13)	0.0121 (12)	−0.0216 (15)
C14	0.0458 (12)	0.0617 (13)	0.0588 (14)	0.0035 (10)	0.0172 (11)	−0.0143 (11)

### Geometric parameters (Å, º)

**Table:**

N1—C1	1.365 (2)	C8—C9	1.375 (3)
N1—C4	1.376 (2)	C8—H8	0.9500
N1—H1A	0.94 (2)	C9—C10	1.391 (3)
C1—C2	1.374 (3)	C9—C13	1.517 (3)
C1—H1	0.9500	C10—C11	1.392 (2)
C2—C3	1.395 (2)	C10—H10	0.9500
C2—H2	0.9500	C11—C14	1.507 (2)
C3—C4	1.379 (2)	C12—H12A	0.9800
C3—H3	0.9500	C12—H12B	0.9800
C4—C5	1.438 (2)	C12—H12C	0.9800
C5—N2	1.275 (2)	C13—H13A	0.9800
C5—H5	0.9500	C13—H13B	0.9800
N2—C6	1.439 (2)	C13—H13C	0.9800
C6—C7	1.395 (3)	C14—H14A	0.9800
C6—C11	1.399 (3)	C14—H14B	0.9800
C7—C8	1.387 (3)	C14—H14C	0.9800
C7—C12	1.509 (3)		
			
C1—N1—C4	108.76 (16)	C8—C9—C10	117.61 (17)
C1—N1—H1A	123.3 (12)	C8—C9—C13	121.47 (19)
C4—N1—H1A	127.7 (12)	C10—C9—C13	120.9 (2)
N1—C1—C2	108.75 (16)	C9—C10—C11	121.61 (18)
N1—C1—H1	125.6	C9—C10—H10	119.2
C2—C1—H1	125.6	C11—C10—H10	119.2
C1—C2—C3	106.88 (16)	C10—C11—C6	119.13 (16)
C1—C2—H2	126.6	C10—C11—C14	119.86 (17)
C3—C2—H2	126.6	C6—C11—C14	121.00 (16)
C4—C3—C2	108.33 (16)	C7—C12—H12A	109.5
C4—C3—H3	125.8	C7—C12—H12B	109.5
C2—C3—H3	125.8	H12A—C12—H12B	109.5
N1—C4—C3	107.28 (15)	C7—C12—H12C	109.5
N1—C4—C5	124.72 (16)	H12A—C12—H12C	109.5
C3—C4—C5	127.83 (17)	H12B—C12—H12C	109.5
N2—C5—C4	125.03 (17)	C9—C13—H13A	109.5
N2—C5—H5	117.5	C9—C13—H13B	109.5
C4—C5—H5	117.5	H13A—C13—H13B	109.5
C5—N2—C6	117.39 (15)	C9—C13—H13C	109.5
C7—C6—C11	120.09 (16)	H13A—C13—H13C	109.5
C7—C6—N2	121.12 (17)	H13B—C13—H13C	109.5
C11—C6—N2	118.70 (15)	C11—C14—H14A	109.5
C8—C7—C6	118.51 (18)	C11—C14—H14B	109.5
C8—C7—C12	120.33 (18)	H14A—C14—H14B	109.5
C6—C7—C12	121.15 (18)	C11—C14—H14C	109.5
C9—C8—C7	123.01 (18)	H14A—C14—H14C	109.5
C9—C8—H8	118.5	H14B—C14—H14C	109.5
C7—C8—H8	118.5		

### Hydrogen-bond geometry (Å, º)

**Table:**

*D*—H···*A*	*D*—H	H···*A*	*D*···*A*	*D*—H···*A*
N1—H1*A*···N2^i^	0.94 (2)	2.05 (2)	2.909 (2)	150.7 (17)

Symmetry code: (i) −*x*+1, −*y*, −*z*+1.
